# The New York Institute of Stomatology

**Published:** 1901-08

**Authors:** 

**Affiliations:** The New York Institute of Stomatology


### THE NEW YORK INSTITUTE OF STOMATOLOGY.

A MEETING of the Institute was held at the office of Dr. Horace J. Parker, No. 27 West Thirty-eighth Street, New York, on Tuesday evening, April 2, 1901.

The minutes of the last meeting were read and approved.

COMMUNICATIONS ON THEORY AND PRACTICE.

*Dr. F. Milton Smith.*—I have one or two practical little things which I use every day in my office, and it has occurred to me that they may be of interest.

First, a glass cube which Dr. H. G. Marshall gave me about ten years ago, which I use to mix cement fillings upon. I find it very convenient because it is frequently necessary to make two mixings of cement without cleaning the slab. This can be done with this cube by simply turning it over. One side has two little places ground out with a carborundum wheel. These are useful in placing a drop of medicament for a treatment.

I should like also to call attention to a set of tools which every laboratory worker will recognize as scrapers. They are of a very small size, indeed much smaller than I had formerly supposed could be obtained. I find them to be of great practical value in plastic filling work, especially in cutting down fillings after they have become hard. I have four of the smallest sizes, and use the back or convex side for packing and burnishing.

*Dr. E. H. Raymond.*—Mr. President and gentlemen, in presenting these casts I do so because they have been so exceedingly interesting to me. They are casts of the teeth of little twin girls ten years of age. They present the most perfect cases of parallel development which I have ever seen under a similar environment. I will call attention to a few of the points. In the upper jaw the canines on the right side are almost fully developed (about two years "ahead of time") and exactly alike. On the left side the points of the canines are visible. The bicuspids are all developed except the left superior second in each case.

The rugæ also are almost identical in number and shape. In the lower jaw there is a perfect development of all except the right twelfth-year molar, and of course the wisdom-teeth. The carious points which have manifested themselves are also alike in each case. The mother tells me that in disposition and temperament they are alike; the color of hair and eyes and the shape of the features are as one. From a dental stand-point I think you will be interested in the two casts.

*The President.*—This is what we would expect to find in the case of twins, yet I have twin patients, young girls, who are exactly the opposite. They are totally unlike in their disposition, their physical make-up, and their teeth. The teeth of one are so frail that they constantly need attention. The teeth of the other are so perfect in structure that they need scarcely any attention whatever.

*Dr. C. O. Kimball.*—I have twin daughters, twenty-one years of age, and seeing these models of Dr. Raymond's calls to my mind the models of their mouths. The history which Dr. Raymond has been giving sounds strangely familiar to me, for the development of my own girls has been almost identical. The minute changes in the mouth, such as the position of decay, have been as he has described, except that they have extended over a longer period of years, so that I have been able to watch the development for a longer time. It is a typical case of twin development, and those who know them know that their faces and figures are almost exactly the same.

*Dr. E. A. Bogue.*—At Dr. Delavan's lecture last Thursday before the dental societies he was asked whether any improvement had been made in the treatment of syphilis in recent years. He replied that there had been no improvement that he knew of in the last fifty years. A few moments later, after a number of gentlemen had left the room, he asked me to repeat what I had just told him, in regard to recent discoveries on this point made at the Pasteur Institute in Paris by Dr. Lisle, an American physician working there. I shall be glad if the President will allow me to state a little more fully here what I tried in a few words to say there.

About two years ago Dr. Lisle thought he had isolated and discovered the microbe of syphilis. I saw his specimens last September, and take pleasure in exhibiting a photomicrograph of them which I have recently received from Paris. Dr. Lisle was to deposit the results of his investigations at the Academy of Medicine in Paris on March 5 last, in order to establish the priority of his discovery, and from this time on Professor Metchnikoff takes personal supervision of the further work of investigations and proving the results of this discovery, all of which goes to show that the matter is seriously considered at the Pasteur Institute. Should the expected results arising from this discovery be attained, the treatment of the disease will undergo a very radical change; and it will be treated by means of antitoxins, much after the fashion that diphtheria, the bubonic plague, and rabies are now treated. In that case hopes may be entertained of a radical cure for the disease, something which no one as yet has dared to predict.

*The President.*—We are to have the pleasure of listening to a paper by Dr. J. C. Bierwirth, of Brooklyn, entitled "Uric Acid in its Relation to Disease, with Special Reference to the Diseases of the Oral Cavity."

*Dr. J. C. Bierwirth.*—When my friend Dr. Parker requested me to read a paper on the subject which I have selected I expressed my doubts as to whether one so purely medical, as this kind must be, would be of interest to your society. However, he was kind enough to tell me to prepare my paper as I saw fit, which I have done. If I am a little deep in chemistry I ask you to pardon me, and I

will simply say that it is necessary for a better understanding of the subject. Dr. Kimball very kindly sent me a list of questions, in reply to a request for some suggestions to guide me in the preparation of my paper. I find out, however, that it will be better to answer these questions in the discussion rather than embody them in the paper.

(For Dr. Bierwirth's paper, see page 534.)

*The President.*—Gentlemen, this splendid paper of Dr. Bierwirth is before you for discussion. We will be very glad to hear from any present who will participate in its discussion.

DISCUSSION.

*Dr. C. B. Parker.*—I first want to thank Dr. Bierwirth for his entertaining and instructive paper, which to me has been very interesting indeed. As to the discussion, I shall be obliged to agree with him so far as I understand the subject. I have never performed any scientific work in this line, but quite a little of what I shall call practical observation. I have been specially interested in a line of work that in a great degree, I firmly believe, has been the result of stasis caused by uric acid. I refer to pyorrhœa alveolaris, caries, and necrosis of the jaw, and where the maxillary sinuses are involved. I believe that if we will try and cover a larger field in our knowledge than the tooth and its immediate vicinity, our patients will soon reap the benefit, and we will be well awarded in spirit.

The small part I shall take in this meeting is to read a few of many cases of record, simply to show where I stand and what I think we are coming to in the treatment of the majority of cases of pyorrhœa. Not to treat them by pushing an instrument clear to the end of the root and sending the patient home sick for the rest of the day, but to use palliative in connection with the important systemic treatment. In that way one will be able to control these cases, even if they cannot be cured promptly. The average treatment that has been given them in the past I know is not necessary, for I have secured far better results with the palliative treatment in conjunction with the uric acid systemic treatment. Now, if what little I have said or in the cases I shall read will but provoke some discussion, I shall be satisfied, for in that we are sure to gain some knowledge.

Case I.—A young lady, Miss A., seventeen years of age, complained of the right superior central feeling sore. I treated with aconite and iodine, and she returned in a few days with several others in the same condition; the festoons between the teeth were beginning to loosen and smell. They were treated as before. Three days later she returned with a slight improvement; the same treatment was repeated. In three days she returned; condition about the same, with the exception of the incisor spoken of, which was beginning to loosen and elongate. A very careful examination was again made, and nothing found around the necks of the teeth. The palliative was continued a week or ten days with no decided improvement. I then advised her to see her physician at once for a physical examination, which was done. A day or two later he telephoned me that he was afraid she was in for a fit of sickness. In a very short time she went through a severe rheumatic fever; all the teeth became so loose and elongated, especially the central, that saving them was despaired of. The uric acid treatment she received cured all trouble in the mouth. Twice in the past eighteen months she has started in with what appeared to be the same condition as the first time, so far as the appearance of the teeth and mucous membrane were concerned. I recommended the systemic treatment in conjunction with the palliative for the teeth and gums, with satisfactory results at once.

Case II.—A physician complained that several of his teeth seemed lame and sore. Every day or two he called for treatment. At first it looked as though we were gaining some headway; then the gums began to swell all along one side of the jaw, two of the teeth becoming quite loose; then a little exudation of serum and pus began to appear. I told him that if he had a patient with the same trouble in the foot he would call it gout and treat for it accordingly. He took my advice, and when I saw him about a week later he had the trouble well under control, it continued to improve from day to day. He told me that he found that his whole system had been filled with uric acid, and in future he would profit by his experience not only with himself, but with his patients as well.

Case III.—Mrs. D. has been treated for pyorrhœa for ten years, about every three months either one tooth or another, losing one occasionally until three or four years ago, when the remaining ones were extracted. She exclaimed then, "Now I will have comfort." Soon after this she had an attack of gout, for the first time, and has continued to have attacks from time to time ever since. I have heard her remark several times since the extraction of the teeth that she did not know which was the worse place to have it, in the jaws or in the feet.

Case IV.—My wife for the past few years has been going through the usual symptoms of a true case of pyorrhœa. In this case there have been from two to four attacks a year, first on one or two teeth, then a change to another location, which keeps you and the patient discouraged. For the past year, with the salicylate of lithia given as soon as the first symptoms appear, I have been able to get complete control of the trouble, and, in fact, keep it under control.

Case V.—A well-known surgeon of our city called to see me, saying that several of his teeth were so sore that it was with great difficulty he could masticate his food. Upon examination I could not see anything special, and treated with aconite and iodine. This condition remained about the same for a few days, and not gaining the headway to suit me, I made an examination with a powerful light made for the purpose, and found that the antrum on one side was badly congested. I diagnosed it stasis from uric acid, and advised salicylate of lithia or any treatment he thought best for that trouble. He followed directions and secured relief at once. I saw him some time later, and he told me that he should continue the treatment, as he was having a little rheumatism.

*Dr. Raymond.*—Dr. Bierwirth has covered the ground so fully, and given us so much for careful study, that I, for one, feel under obligations to him for his valuable contribution, bearing upon a subject of such importance to us. Patients who come under our care with marked uric acid diathesis not only require our mechanical skill, but the ever-present enemy, uric acid in the blood, must be attacked through general systemic treatment if we would save the teeth at all. In cities and towns where people of this peculiar temperament get little pure air or exercise, and are large eaters, it is almost impossible to prevent the erosion around the necks of their teeth. If the kidneys are unable to carry off the uric acid which is found in the liver, the blood will naturally convey it through the system. You will find on the surface of the skin little pustules containing uric acid. An examination will reveal crystals of this acid. If you will put the end of your tongue on the inside of your lip, you will feel very plainly the mucous glands or follicles. It is well understood that these glands are receptacles for uric acid, and this passing through the ducts of the mucous membrane on to the necks of the teeth causes much of the erosion which we meet with by its solvent action upon the phosphates and carbonates of the enamel. Our duty in such cases is to prescribe constitutional and dietary treatment. Pure air and golf will be better than massage and poker, which many deem apparently an efficient substitute for healthful exercise. Cervical decay, however, is not always due to the action of uric acid. I have seen marked cases where there was no tendency to uræmia in any of its phases.

*Dr. J. W. Canady.*—It is a very large subject and one on which a great deal can be said. I believe with Dr. Parker that the treatment of these obscure cases of erosion should be more systemic than instrumental.

*Dr. Charles J. Meeker.*—There is one question that I would like to ask Dr. Bierwirth. I may ask it by citing a case in my own practice. It is that of a lady thirty years of age, who has been in my charge for twenty years and has always been in excellent health. She has within the last six months developed enormous quantities of tartar. Is this any indication of uric acid poisoning? How would Dr. Bierwirth look at this from a physician's stand-point?

*Dr. Bierwirth.*—I think that in this case there would be indicated perhaps a disease of the glands rather than any constitutional disturbance. Certain forms of anæmia may also be the cause. There are so many conditions which might cause this trouble that it is hardly safe to say which was the cause in this particular case.

*The President.*—We have been much favored and instructed by this paper of Dr. Bierwirth's, and I am sure that we as well as the larger number who will read the report of this meeting will profit by what he has said. It is a field in which we are happily beginning to learn, and of course our minds revert naturally to the paper which Dr. Michaels, of Paris, sent us somewhat over a year ago, and to the recently published synopsis of a paper that he read before the International Congress at Paris last summer. Dr. Michaels has attributed certain variations of the chemical composition of the saliva to the uric acid diathesis, and erosion of the teeth and other lesions are due, in his opinion, to abnormal quantities of the sulphocyanides and other constituents. This paper of Dr. Bierwirth's is undoubtedly going to be of great help to us in understanding this subject. I was impressed, in referring to Schafer's recent work on physiology, by the statement that the amount of sulphocyanates in human saliva varies from 0.014 to 0.00016 per cent. In the "American System of Dentistry" the analysis of Bidder and Schmidt place it at 0.006 per cent. So we see what a delicate undertaking Dr. Michaels has had in hand when seeking to determine for individual patients variations from the normal amount of this constituent of their saliva.

*Dr. Bierwirth.*—I would like first to answer the questions which Dr. Kimball has sent me.

1. Pyorrhœa alveolaris, chemical erosion of the teeth, decay of teeth, and recession or atrophy of the gums have all been observed to be associated with the uric acid diathesis. Yes.

2. Dr. Peirce, of Philadelphia, has said that hard deposits form on roots of teeth, composed of urates, before any opening connects the point of deposit with the surface.

I take exception to this, as I contend that it is impossible to know whether there is a microscopical opening or not, and also on the ground that it is inconsistent, but mainly for the former reason.

3. Dr. Michaels, of Paris,, claims that dental lesions, erosion, and caries are results of discrasia. Shown by analysis of saliva as well as of urine. He says where ammoniacal salts exist in saliva in excess of sulphocyanides dental caries is in active progress. When sulphocyanides are in excess of ammonia salts, caries is checked, but in this condition, if potassium is in combination with sulphocyanides, erosion takes place. In a healthy state of the system he says the ammonia and sulphocyanides are in equal proportions in the saliva and in small quantity. Excess of sulphocyanides is characteristic of hyperacidity (arthritism).

I believe this is fishing for the unknowable. The saliva is an alkaline fluid which is poured out in enormous quantities. It contains normally always the sulphocyanide of potassium. All the analyses that I have ever seen have contained a certain amount of this salt. Now, this has been variously given as from 0.05 per cent. to 0.006 per cent. A great deal depends upon the means by which the saliva is collected. It would seem, in that the sulphocyanides are always present, that if this salt causes erosion all the teeth in the mouth would soon be lost.

4. Would an analysis of saliva furnish a basis for diagnosis of general conditions?

I do not believe it. I wish to modify that a little. I do not believe that the saliva has any other function in the human system except to act on raw starch and to act as a lubricant. There has been a great deal of controversy as to whether the ptyalin has any action after it reaches the stomach. I believe that the latest authorities state that in the stomach its action is merely inhibited and that it is continued in the duodenum. I cannot bring myself to the belief that the saliva has anything to do with the caries of the teeth. If so, we should have a great deal more than we do have. It is a perfectly bland fluid. It may be changed more or less by disturbances of the stomach. I think the way most chemical processes are set up which destroy tooth-structure is by the retaining of food between the teeth, producing a focus of sufficient strength to attack the enamel of the dentine below the enamel. I simply reason this from the stand-point of a physician and a chemist. I believe this acts in combination with a lowered resistance of the tooth itself, although this is not necessary and the process may be purely chemical, as we all have seen apparently perfectly healthy persons who take good care of their teeth and yet every tooth has a filling in it.

And so I think, in searching for a logical and scientific cure for caries of teeth we must always bear in mind that there is a lowered resistance in the tooth and a local focus, which has acted upon that spot.

*Dr. Kimball.*—I wish to express, on behalf of the Executive Committee and the Institute, our pleasure and thanks to Dr. Bierwirth for coming over from Brooklyn to read us this excellent paper, and I move you, Mr. President, that the thanks of the Institute be most cordially extended to our friend. Seconded and carried.

I am instructed by the Board of Directors to make a report to the Institute.

REPORT BY BOARD OF DIRECTORS.

At the suggestion of Dr. Benjamin Lord, the first president of the Institute, and largely through his personal influence, a Special Fund has been established under the control of the Board of Directors of the Institute, for the purpose of promoting the advancement of the profession along lines of scientific research or investigation. This fund has been added to by some of the members until it amounts at the present time to eleven hundred dollars.

Contributions of any amount to this Special Fund will be accepted with thanks, and suggestions will be welcomed as to the most effective use that can be made of it for the purpose stated.

*Dr. E. A. Bogue.*—I would like to ask Dr. Bierwirth one or two questions:

1. Whether he would consider the condition which he has so graphically described to us in his paper as having any bearing whatever on diabetes?

2. Whether it would be possible for the liver to produce more sugar that was wanted and eventually discharge it through the salivary glands into the mouth, if not in the form of sugar in the form of glycogen?

*Dr. Bierwirth.*—In answer to the first, I hardly think so. As a rule, in diabetes there is no congestion of the mucous membrane, although congestion may come subsequently. Of course, diabetes may cause it, but I doubt it very much.

2. I do not believe that this is so. I have too much faith in the perfect balance of nature. I think if anything of this kind could occur it would be readily recognizable, and think it would have been found out long since. I do not think much faith should be placed on laboratory experiments causing erosions, where the teeth are taken out of their natural environment.

*Dr. Bogue.*—There is one other question, whether either of these substances, sugar or glycogen, should they come into the mouth in this manner, could in combination with the ptyalin form an acid?

*Dr. Bierwirth.*—I do not believe that ptyalin has any influence on sugar in any way. Chemically considered, I do not think this is possible.

Adjourned.

FRED. L. BOGUE, M.D., D.D.S.,
*Editor The New York Institute of Stomatology.*

